# Managing Floral Resources in Apple Orchards for Pest Control: Ideas, Experiences and Future Directions

**DOI:** 10.3390/insects10080247

**Published:** 2019-08-11

**Authors:** Annette Herz, Fabian Cahenzli, Servane Penvern, Lukas Pfiffner, Marco Tasin, Lene Sigsgaard

**Affiliations:** 1 Julius Kühn-Institut, Institute for Biological Control, Heinrichstr. 243, 64287 Darmstadt, Germany; 2 Department of Crop Sciences, Research Institute of Organic Agriculture (FiBL), Ackerstrasse 113, 5070 Frick, Switzerland; 3 INRA, Centre de Recherche PACA, UR Ecodeveloppement, 84914 Avignon, France; 4 Department of Plant Protection Biology—Unit of Integrated Plant Protection, Swedish University of Agricultural Science, P.O. Box 102, SE-230 53 Alnarp, Sweden; 5 Department of Plant and Environmental Sciences, University of Copenhagen (UCPH), Thorvaldsensvej 40, DK-1871 Frederiksberg C, Denmark

**Keywords:** biological control, ecological infrastructure, fruit growing, functional biodiversity, integrated pest management

## Abstract

Functional biodiversity is of fundamental importance for pest control. Many natural enemies rely on floral resources to complete their life cycle. Farmers need to ensure the availability of suitable and sufficient floral biodiversity. This review summarizes 66 studies on the management of floral biodiversity in apple orchards, published since 1986. Approaches followed different degrees of intervention: short-term practices (mowing regime and weed maintenance, cover crops), establishment of durable ecological infrastructures (perennial flower strips, hedgerows) and re-design of the crop system (intercropping, agroforestry). Although short-term practices did not always target the nutrition of natural enemies by flowering plants, living conditions for them (alternative prey, provision of habitat) were often improved. Perennial flower strips reliably enhanced natural enemies and techniques for their introduction continuously developed. Resident natural enemies and their impact in pest control reacted positively to the introduction of a more diversified vegetation, whereas the response of very mobile organisms was often not directly linked to the measures taken. A careful selection and management of plants with particular traits exploitable by most natural enemies emerged as a key-point for success. Now the elaborated design of such measures needs to be adopted by stakeholders and policy makers to encourage farmers to implement these measures in their orchards.

## 1. Introduction

Apple orchards are perennial crop systems, which may dominate the environment due to their area-wide cultivation. Commercial cropping systems involve the cultivation of intensively managed dwarf-trained trees, with a high input of pesticides, herbicides, and fertilizers. For this reason, their management can seriously affect the environment, but in particular local biodiversity within the orchard itself [1,2]. A more traditional, low-input practice is the cultivation of a mixture of various high-stem apple tree varieties, which are scattered in meadows and managed without pest or vegetation control [3,4]. Also, organic fruit growing is considered to be less intensive [5,6]. Nonetheless, proper tree training with selected cultivars, regular use of biological plant protection products, organic fertilizers and vegetation management are common treatments to obtain reliable and satisfying yield in organic production as well [7]. 

Compared with annual arable crop systems, perennial orchards offer a habitat for a more diverse community of organisms with different ecological needs [7,8,9,10]. Orchards provide different strata of a permanent nature and a typical biocoenosis can evolve and survive, unless pesticides and other management practices substantially harm these organisms [2]. Particular functional groups of organisms provide essential ecosystem services, which are important for sustainable fruit production [2,8]. Maintenance and promotion of this functional biodiversity can be the target of tailored management practices. 

In addition to the cultivated fruit trees in the orchard, other plants contribute to the diverse environment and provide nutritional or structuring functions. Hedges around and within the orchard often serve as windbreaks [11], and also constitute essential ecological infrastructures providing nesting sites and nutrition resources for birds, mammals, and arthropods [12,13]. Flowering plants and grasses, wild or sown, between or within tree rows prevent soil erosion and loss of water capacity. They can also serve as host plants for non-pest herbivores that are alternative prey for natural enemies as well as providers of nectar and pollen for flower-visiting insects. Intercropping with flowering crops can contribute to floral diversity too. In the view of ecosystem services, plant and especially floral diversity are considered as a bottom-up system to favor pollinators and natural enemies [14,15,16]. In the last 30 years, many studies were conducted on the design, effects and management of floral and structural resources provided by ground cover vegetation, cover crops, hedges or tree composition in orchards. Previous reviews collected available scientific evidence and evaluated advantages and disadvantages of the various strategies tested [17,18]. Simon et al. considered 30 case studies, published in 22 articles until 2008, on the manipulation of orchard plant diversity with the aim to improve pest control [2]. In at least 16 cases, a positive effect was found on the control of one or more of the following taxa: aphids, leafhoppers, spider mites, and tortricids. Research on this topic has continued and recently published articles have reported new approaches and can give additional insights. Accordingly, we conducted a systematic review of publications in the scientific literature since 1986, describing various techniques to increase local floral diversity in apple orchards that could be implemented by growers. We focused explicitly on the management of floral resources within orchards with the aim of favoring beneficial arthropods and providing better pest control in apple production. 

## 2. Materials and Methods 

We performed a literature search in ISI Web of Knowledge (Databases Web of Science, CABI Abstracts) with the following search string: 

TOPIC: [((((apple *) AND (Orchard *) AND (“cover crop *” OR “service crop *” OR “flower * crop *” OR “semi-natural” OR “floral *” OR “flower strip *” OR “flower margin *” OR “non-crop flower *” OR “non crop flower *” OR “intercrop *” OR “inter-crop *” OR “hedge *” OR “weed *” OR “vegetation” OR “wild plant *” OR “agro-forestry” OR “agroforestry” OR “* diversity”) AND (“pest *”)))), Timespan: 1987–1996, 1997–2006, 2007–2017, 2018 (record date: 16 May 2018)], resulting in more than 1600 citations. Titles were checked, duplicates were removed and only full papers published in English language, but without any geographic restriction were included. Furthermore, we added single references, which had been collected by authors for a literature collection available on the information portal “EBIO-Network” [19] and one publication on our own field study in the CoreOrganic Plus research project EcoOrchard, published recently [19]. 

Following this procedure, we found 702 references. Abstracts were screened in more detail in order to decide if they match the general question: “Managing floral resources for pest control in apple orchards”. Only primary studies from empirical research, focusing on natural enemies and their ecosystem service “pest control” were considered for further reviewing. Articles presenting studies at the landscape level or dealing mainly with other ecosystem services like pollination, soil improvement and water regulation were not included. Suitable articles were classified according to their content into particular categories of practices (mowing regime and weed maintenance, cover crops, flower strips, hedgerows, intercropping and agroforestry). 

Sixty-six studies were included for the final data analysis. Studies were analyzed according to the following criteria (1) level of study (scientific/research vs. practice), (2) region, size and duration of study, (3) farming system, (4) floral resources created/ manipulated, (5) effect on target pests (no. 1, 2, 3, etc.) (6) effect on abundance of beneficials (which?), (7) effect on species richness of beneficials (which?), (8) effect on fruit damage and yield, (9) any unwanted effects. According to our evaluation, the collected set of studies did not allow us to conduct a quantitative synthesis using statistical techniques (i.e., a meta-analysis). The main reasons are: (1) variations in study design (split-plot, no replicates or many replicates, use of pesticides or not, commercial fields versus experimental fields, duration of study, etc.); (2) differences in the dependent variables measured (insect densities or counts in various kinds of situations or traps (most studies—but different species/groups were considered, probably due to different expertise of the researchers involved), pest control level (few studies), fruit damage or fruit yield (very few studies); (3) differences in data analysis and statistical parameters calculated and presented (for instance, older studies used different statistical approaches than more recent studies, which frequently applied a sophisticated toolbox of statistical methods); (4) occurrence of “clustered” studies, which are not really independent from each other, because studies on a particular practice were often conducted by the same research team in consecutive trials. Therefore, we present the documented evidence regarding our target question in the form of a narrative synthesis of the main findings and conclusions. 

## 3. Results and Discussion

**Figure 1:**
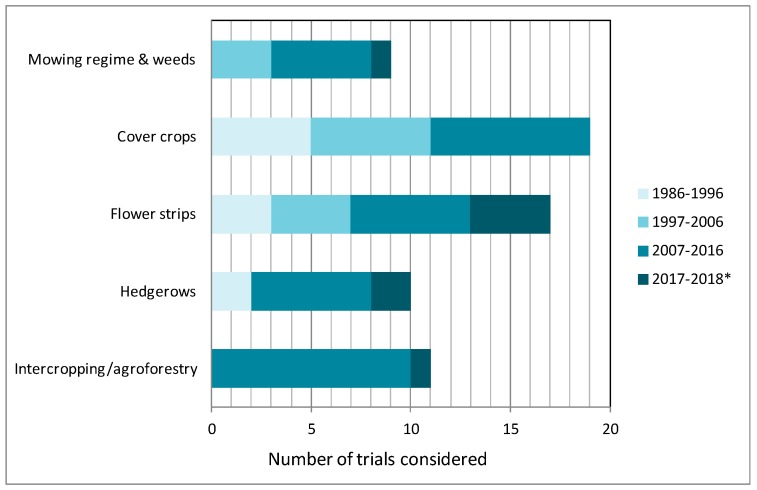
Number of trials reported in publications considered in the review. Studies are shown in relation to the publication period and the different categories referring to the intervention. A few studies are from pear orchards. *: record date 16 May 18 plus one article from 2019.

In general, few matching references were found per category (Supplementary Material: Tables S1–S5). There has been an increase in the number of relevant studies over the last decade (Figure 1). Similar plant species were considered that offer resources in flowers with a short corolla or provide extrafloral nectaries (Table 1). Here, pollen and nectar rewards are easily exploitable by flower visitors with short-tongued or unspecialized mouth parts such as many beneficial flies (Syrphidae, Tachinidae, and others), lacewings, parasitoid Hymenoptera and beetles [14,20,21,22,23,24]. 

Nearly all of the studies spanned an experimental period of not more than one to three years and were often conducted in experimental fields. This allowed a simple manipulation of the particular management; e.g., by tailoring the pesticide application regime or other necessary cultivation techniques. Few studies took place in commercial orchards and these included conventional, IPM, organic and cider apple orchards. Six articles mentioned similar trials in pear and were also considered, because they share some target pests with apples (e.g., *Cydia pomonella*, leafrollers). In a pragmatic approach, we have summarized the main findings for the different categories of practices and ordered these sections in increasing degree of intervention into the general orchard management. 

### 3.1. Adaptation of Mowing Regime and Weed Maintenance 

Increasing plant diversity and enhancing the availability of floral resources may be achieved by adjusting the mowing/mulching regime of the spontaneous orchard ground cover, thus allowing the development of complementary plants such as “weeds” and their flowering. Nine studies addressed this kind of ground cover manipulation by examining the effect of modified mowing frequency on various groups of insects (beneficials and pests). 

Less disturbance of the ground vegetation can improve habitat conditions for soil-dwelling and ground-cover living natural enemies of apple pests. A ground cover of *Trifolium repens* L. and other herbs and grasses in unmown plots conserved populations of the ground beetle *Chlaenius micans* (Fabricius) in a Japanese apple orchard [25]. Whereas the introduction of floral resources was not the aim of these trials, authors assumed that more pollen was available in the unmown plots. As a consequence, the predatory, but also pollen-feeding mites (*Amblyseius tsugawai* Ehara and *Typhlodromus vulgaris* Ehara) were more frequent in unmown plots, both in the understory and on apple leaves, and reduction of spider mites was recorded here [26,27]. Higher arthropod species diversity and abundance (including spiders and tortricid moths of secondary importance) on apple trees in not or less mulched orchards in comparison with frequently mulched plantations were observed in Southwestern Germany [28,29]. Plant diversity was higher and weed strips sown with a few species (*Raphanus sativus* L., *Sinapis arvensis* L., *Coriandrum sativum* L.) in two orchards also had a high diversity of natural weeds. In general, floral diversity was higher in spring and decreased during summer. Sown species could compensate for the lack of flowering plants in the spontaneous vegetation [28]. More spiders in the tree canopy and increased densities of predators and parasitoids in the groundcover were found in a study in a (pear) orchard in the United States [30]. Here, availability of “broadleaf plants” and alternative prey in the habitat increased by less frequent mowing of the spontaneous vegetation (once per month or only once per season) in comparison with the common practice of biweekly mowing. Plants were in blossom in the less frequently mown orchards over the entire season, but attractiveness of blooming on natural enemy abundance was not monitored. Three mowing regimes were applied in an apple orchard in Southeastern France [31]: tall (no mowing), medium (20 cm height maintained by bi-weekly mowing) and short (5 cm height maintained by weekly mowing). The spontaneous vegetation in the inter-rows consisted of grasses and several herbs. No effect of ground cover management on the numbers of earwigs or spiders in cardboard traps at tree stems was found. Pest control effects were measured by estimating predation rate on sentinel codling moth eggs. Predation rate on these sentinels increased from April to July, but were lower in the tall vegetation plots later in the season. The authors concluded that this pattern could be due to the presence of alternative prey resources in the grass and/or trees, although not directly measured. Again, this study considered the response of natural enemies to increasing plant cover height in the inter-rows, but not explicitly to the blooming of these plants. 

In contrast, the possible effect of flowering forbs on natural enemies was the major objective in the study by García and Miñarro [32]. They documented spontaneously grown floral resources both in tree lines and inter-rows in nine cider apple orchards in Northwestern Spain without any experimental manipulation of the standard mowing regime. Observed flower-visiting insects, including aphidophagous hoverflies, were most diverse and abundant in those orchards with highest species richness of flowering plants. Such positive effects were also found for predatory Coleoptera (Coccinellidae, Carabidae) and Hemiptera (Anthocoridae, Nabidae, Lygaeidae). In Australia, potential effects of local floral richness (mainly *Taraxacum* spp., *Trifolium repens* L., *Veronica* sp., *Plantago* sp. and various Brassicaceae) in the orchard understory on insect communities was evaluated on focus trees within six apple orchards (conventional and organic) [33]. Local flower richness was positively related with observed natural enemy richness (syrphid flies, parasitoid and vespoid wasps). These two reports clearly indicated the suitability of floral resources provided by the spontaneous orchard vegetation in monocrop orchard systems. 

Thus, less mowing in inter-rows can make habitat conditions more suitable for natural enemies, provide alternative prey, and will favor the development of flowering forbs. More resident arthropods like spiders and predatory mites responded by increased numbers even in the tree canopy, and blooming in the understory also attracted mobile insects searching for flower rewards. Such a practice should be very attractive to growers, because no additional investment is required and working load and tractor hours may be reduced as well. Further research on adjusted mowing regimes needs to relate the function of the spontaneous vegetation and its flowering to the activity of natural enemies more clearly. One major issue is the connection and migration of natural enemies between groundcover and tree canopy. For example, (alternate) mowing just before outbreaks of aphids may be recommended to foster the migration of predators to trees. But evidence for better pest control is still sketchy and it is of particular relevance to investigate potential impacts on biological control and improved fruit health after such manipulations. 

### 3.2. Cover Crops 

Cover crops are plants primarily planted to improve conditions for the main cash crops, and are not meant to be harvested. The main practical reasons for establishing cover crops in orchards are soil fertilization, prevention of soil erosion and weed suppression. The selected plant species should not be the source of pests or compete with the crop for water, nutrients, pollination and light. They need to establish easily and quickly, building up a cover for weed suppression. Cover crops should not require any particular management interfering with the necessary management of the main crop. Typical cover crops are uniform; there exist cover crop cultivars that are the product of intensive efforts of selective breeding and seed production. Weed control and tree nutrition by cover crops are important aspects in orchard systems, especially in organic production, where synthetic fertilizers and herbicides are not allowed [34,35]. Besides these aspects, cover crops may also contribute to better pest management due to altered host plant nutrition or microclimate in orchards as well as by providing essential resources (shelter, alternative prey, nectar, and pollen) to benefit natural enemies [17]. The selection of suitable plants must take into account the natural enemies’ needs, such as the inclusion of plants with disk flowers and open nectaries [14,20,21,22]. Research on introducing cover crops as floral resources into apple orchards has quite a long history and comprises many of the studies reviewed here (Figure 1). Typical flowering cover crops like buckwheat (*Fagopyrum esculentum* Moench), Phacelia (*Phacelia tanacetifolia* Benth., alfalfa (*Medicago sativa* L.) or other Fabacaeae including white clover (*T. repens* L.) alone or mixed with rye were tested in a number of studies performed mainly in experimental orchards using a small plot-design [36,37,38,39,40,41,42,43,44], (Table 1). 

**Figure 2:**
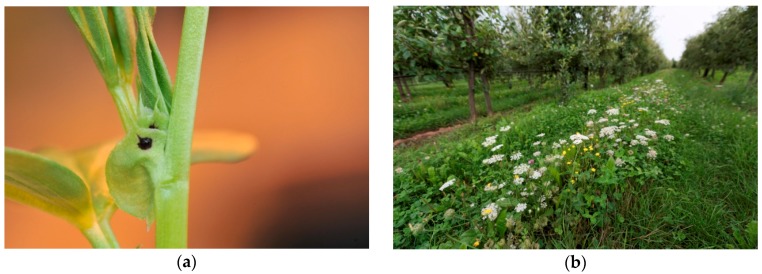
Floral resources to promote natural enemies in apple orchards. (**a**) Faba bean with extrafloral glands situated in the black spot on the Stipulae. Faba bean was experimented as cover crop. (**b**) Flower strip composed of perennial plant species between tree rows. Fotos: Annette Herz, JKI Darmstadt © (**a**), Simon Feiertag, JKI Darmstadt © (**b**).

Legumes, like faba bean (*Vicia faba* L.), other *Vicia* species, clover or alfalfa grown as a cover crop in orchards are often introduced because they may provide several ecosystem services like fertilization, prevention of soil erosion, alternative prey (*Aphis fabae* Scopoli) and flowers. [17,34,35]. Moreover, vetches (e.g., *Vicia sepium* L. and *Vicia sativa* L.,) offer carbohydrates in plant sap from special structures, so-called extrafloral nectaries (EFN) [45] in addition to floral nectar (Figure 2a). These EFN glands are assumed to deliver resources to attract predators in order to create some kind of indirect defence against pests [46] and they can be easily exploited due to their exposed nature. For instance, parasitoid Hymenoptera are known to visit EFN of faba bean [47]. Use of faba bean EFN also increased the longevity of *Ascogaster quadridentata* Wesmael, a parasitoid of the codling moth in the laboratory [48]. Altieri et al. planted *Vicia* sp. and various clovers in apple orchards and found higher abundances of beneficials, especially spiders and ants [49]. They also observed higher predation on cards with sentinel eggs and larvae, lower leafhopper and aphid densities and lower fruit damage by codling moth in the experimental plots with cover crops in comparison with disked or herbicide-treated parts of the orchard. In a four year-study in a Washington state apple orchard, pest densities, natural enemies and fruit damage or yield were examined in plots with standard grass cover versus alfalfa cover [50,51]. No clear pattern could be observed in pest damage (codling moth, leafrollers), but alfalfa cover did not create any unwanted effects. Natural enemy densities increased over time in both treatments [51]. Leafroller parasitism was “modestly encouraged” by alfalfa [50]. Alfalfa was not flowering in this experiment. Eventually, benefits from alfalfa for natural enemies in this study would thus have been from alternative prey and any extrafloral nectaries [46,52]. In another study in China, enhanced populations of the anthocorid *Orius sauteri* (Poppius) in the cover-cropped area (alfalfa and *Lagopsis supina* (Steph. ex Willd.) (Lamiaceae)) of an apple orchard kept spider mite densities below the economic threshold in comparison with the vegetation free control area [44]. A four-fold lower density of the green apple aphid (*Aphis pomi* De Geer) was found on apple trees accompanied by a mixture of white clover and grass as cover crops in comparison with plots treated with herbicides, covered with plastic mulch or rye [39]. However, according to the authors, the lower aphid density was probably due to the lower nitrogen content in apple leaves in the clover-grass treatment rather than to higher pest control. 

Certain nectar-rich flowering plants are known to promote survival and fecundity of natural enemies as revealed in many laboratory studies. Hence, their suitability as cover crops in apple orchards was also tested in the field (Table 1). In a field trial in New Zealand, coriander and buckwheat were planted in uniform strips beside apple trees [41]. In these plots, parasitism of previously exposed sentinel eggs and larvae of the light brown apple moth *Epiphyas postvittana* (Walker) by the parasitoid *Dolichogenidea tasmanica* Cameron was higher in comparison with herbicide-treated control plots. In following studies, more *D. tasmanica* were also documented in buckwheat, faba bean or Alyssum (*Lobularia maritima* L.) plots than in the control and, again, these flowers also resulted in higher parasitism on sentinel host larvae [40,42]. In the 2006 study, also the parasitism of eggs and larvae of the natural populations of the light brown apple moth was higher and fewer pupae of the pest were found in plots with buckwheat, phacelia and Alyssum [40]. Laboratory trials revealed that longevity of male and female parasitoids was up to five times longer when having access to faba bean, buckwheat, coriander, alone or in combination, and Alyssum in comparison with water, thus confirming the value of these nectar plants for the parasitoids [40,41]. In a more recent study [53], attractiveness of Alyssum plots for natural enemies was estimated in an experimental apple orchard in Washington State, USA. Moreover, the regulation of artificially introduced woolly apple aphids (*Eriosoma lanigerum* (Hausmann)) on potted apple trees was compared within Alyssum and grassy plots. Only one week after exposure, aphid densities were significantly lower on trees adjacent to Alyssum than in control plots and these differences were maintained for several weeks. Immunomarking revealed that natural enemies regularly moved from Alyssum to the surrounding orchard. The cover crop attracted many natural enemies (Syrphidae, predatory bugs, and spiders) and some of them were certainly responsible for the rapid suppression of the pest on the introduced apple trees. 

Even when carefully selected for their benefits to natural enemies (Table 1), cover crops can also carry unwanted side effects in the orchard [36,54]. For example, various cover crops from different plant families (Apiaceae: *Ammi majus* L., *Foeniculum vulgare* Mill.; Asteraceae: *Cichorium intybus* L., *Achillea millefolium* L.; Brassicaceae: *Sinapis alba* L.; Polygonaceae: *F. esculentum*; Fabaceae: *Trigonella foenum-graecum* L.) in comparison with the “grassy” control cover (*Lolium* sp., *Festuca* sp.) were established in three commercial orchards in Australia [54]. Counts of natural enemies (parasitoids, ladybird beetles, lacewings) in the apple tree canopy did not differ between cover crop treatments, but in some crops a higher pest infestation, fruit damage and russetting (e.g., when white mustard (*S. alba*) grew very tall) as well as a lower weight of harvested apples was found. This could probably be due to higher competition for water and nutrients. Voles can also create serious problems to apple trees in cover-cropped orchards, because tall vegetation between trees might favor their population development. The effect of inter-row grown buckwheat and other plant species on vole density was studied in Poland [55]. Buckwheat or mustard had no toxic or repellent effect on voles and an increase in vole density could only be prevented by cutting the same cover plants during the summer. 

Cover crops increased numbers of predatory arthropods such as spiders or anthocorids and also specific parasitoids. Sometimes, even improved control of target pests or introduced sentinel prey or hosts was demonstrated. Effects were more reliable in this respect when the cover crop species were selected to increase the fitness of natural enemies by providing high-value flowers. However, more natural enemies did not always lead to sufficient reduction of pest numbers or lower fruit damage. Unwanted bottom-up effects of cover crops like competition with the main crop, favoring of some insect pests and promotion of diseases or voles as a consequence of changed microclimatic conditions probably masked the direct positive impact of increased floral resources on natural enemies and their ecosystem service. Future research should target vegetation management of high-rewarding cover crops like Alyssum in a way that is compatible with and not harmful to orchard practice. 

### 3.3. Annual and Perennial Flower Strips 

Flower strips are longitudinal structures within or around the crop system. They are established by removing any existing ground vegetation (spontaneous vegetation, green cover) and subsequent sowing a mixture of selected plant species. These can be annuals, intended to be self-seeding or annually re-seeded, or perennial plant species sown with the aim of a long-lasting establishment (Figure 2b). Such flower strips are sources of food to beneficial arthropods in the form of pollen, nectar and alternative prey. They can also provide overwintering protection and shelter when they remain uncut or are not cut too short. This may be especially true for perennial flower strips [56]. 

Seven studies on effects of flower strips for pest control in apple orchards were published during the early 20-year period of our review (1987–2007). Research on this topic boomed during the last decade (2008–2018), resulting in nine studies plus the one published just recently by Cahenzli et al. within the framework of the research project EcoOrchard [57]. The effect of flower strips has most often been assessed on sap-feeding insects, principally aphids. One of the first studies was an unreplicated trial in a Swiss orchard, one half sown with flower strips, the other without [58,59,60]. The flower mixture included selected perennial as well as annual species. The focus of the study was on the control of aphids. The rosy apple aphid *Dysaphis plantaginea* Pass., for instance, is a major pest in apple, requiring regular insecticide applications even in organic production [61]. It was found that *D. plantaginea* and *A. pomi* on trees accompanied by the flower strips were significantly reduced in comparison with trees without flower strips and that the density of spiders and spider webs were two to three times higher in the orchard part with flower strips than in the control area [60]. Spider webs catch flying aphids migrating back to orchards from summer hosts and therefore prevent aphids from establishing the next generation. Indeed, the authors could show that a lower density of aphids in autumn resulted in a lower number of aphid winter eggs laid and, thus, a lower number of fundatrices in the following spring [60]. This finding is in line with a recently published study that was performed over six growing seasons in an experimental, insecticide-free apple orchard, again in Switzerland [62]. Here, flower strips between tree rows, flowering plants within apple tree rows, and flower strips connecting adjacent hedges with the apple trees were created, also enabling the colonization of orchards by spiders [63]. A simultaneous increase in web area of Araneidae and Tetragnathidae decreased the number of aphid fundatrices in spring [62]. Fruit damage by aphids was also decreased with a higher abundance of web-building spiders the previous autumn. An increase of 10% (Araneidae) or 26.5% (Tetragnathidae) in web area decreased aphid fruit damage by 10%. Though some spiders can directly benefit from nectar or pollen provided by flower strips [64,65], the more important contribution to higher spider abundance may come from higher habitat complexity and augmented alternative prey availability [10,60]. Beating samples indicated three to four times more insects of an indifferent status in the orchard part with flower strips as compared with the control, thus increasing the available prey for the spider populations [60]. 

Natural enemy groups, including those considered exclusively predatory can directly use nectar and pollen provided by flower strips [23,65]. However, in field studies it may be difficult to distinguish between the value of pollen and nectar versus the value of alternative prey and habitat to these predators. Indeed, these elements may complement each other. For instance, in the early Swiss study, not only were more spiders found on trees in the flower strip part than in the control, but numbers of predaceous Heteroptera, Coccinellidae, and Chrysopidae [58] and species diversity of predacious groups increased as well [59]. Higher numbers of beneficial arthropods were also reported in perennial flower strips in a plot trial (one orchard) in Czech Republic [66]. Significantly more Syrphidae (>three times more), Tachinidae (>40 times more) and Ichneumonidae (almost three times more) were counted in flower strips in comparison to the grass cover control. Other natural enemy groups (Coccinellidae, Anthocoridae and others) were documented only in small numbers, but this was probably a consequence of the chosen monitoring technique by visual observation in transect walking. In a French study, parasitoid Hymenoptera were attracted by a range of sown flowering plants in an apple orchard, but no key natural enemies of the main target pest (codling moth) were observed among them [67]. 

The promotion of not only individual taxa, but rather the whole complex of aphidophagous and generalist predators, appears to be more promising to achieve sustainable aphid control throughout the season [68]. Indeed, flower strips composed of *Centaurea cyanus* L., *Silene vulgaris* (Moench), *S. latifolia alba* Poir. and *Achillea millefolium* L. increased the overall natural enemy populations in a recent two-year study in Northern France [69]. Also, a positive impact of the flower strips in reducing *D. plantaginea* densities could be demonstrated and both effects decreased with distance from flower strips. In a pan-European study in organic apple orchards, perennial flower strips with native plant species sown between tree rows not only generally increased plant diversity as compared with the spontaneous orchard vegetation, but also clearly boosted flowering forbs, which are known to promote functional biodiversity [70]. As a result, flower strips significantly increased the number of Syrphidae, Chrysopidae and generalist predators (Anthocoridae, Miridae and spiders) on apple trees and in particular natural enemies in *D. plantaginea* colonies [57]. This led to a slower increase in *D. plantaginea* populations in plots with flower strips as compared with control plots and thus reduced fruit damage after the second fruit drop. 

Although flower strips can promote aphidophagous natural enemies, general aphid control through increased populations of natural enemies by flower strips is not guaranteed. For instance, Kienzle et al. observed higher numbers of Coccinellidae, Syrphidae, Anthocoridae, the predatory gall midge *Aphidoletes aphidimyza* (Rondani) and aphid parasitoids in commercial orchards with perennial multi-species flower strips compared with those without in Southwest Germany (Neckar valley) [71]. They also found more syrphid eggs and larvae in green apple aphid colonies on potted, artificially infested apple trees placed in the flower strip-equipped orchards. In the second experimental region of this study (Lake Constance), only Syrphidae were observed in higher densities in the orchards with flower strips. Natural aphid infestation was generally very low, because Neem-based plant protection products were applied as common practice and no differences were found between orchards with and without flower strips. Contradicting results for aphid control by flower strips were experienced in another German study performed over three years with a similar set-up as in the Swiss study [58,59,60] to replicate the positive results [72]. A similar plant mixture in the flower strip was used, but in a newly planted orchard and the flower strips were cut before winter. Higher infestation levels of *D. plantaginea* in the flower strip treatment as compared with the control were found, while lower densities were found of *A. pomi*, which has its peak usually four weeks later than *D. plantaginea*. The authors suggested that the occurrence of *D. plantaginea* before blooming and the cutting of flower strips, thereby removing winter habitats for predators, might explain the different results for the two aphid species. 

Apart from the positive effect of spiders in autumn on aphid control in spring, Cahenzli et al. found no significant effect of more mobile aphid specialist predators such as Coccinellidae and Syrphidae on fruit damage [62]. In contrast to the positive effects of spiders on pest control found by the Swiss studies [60,62] and despite higher spider diversity in flower strips as compared with bare soil and grass only plots, no link to a reduction of pests was found in a trial in a conventionally managed orchard in Hungary [73]. However, effects of spiders, especially of web-building spiders, on wingless, non-migrating pests may be limited. Ambiguous effects of flower strips on pest control in this system were also found on other interactions between natural enemies and pest insects [74]. Regarding natural enemies, Phytoseiidae, parasitoids, beetles and the green lacewing were more numerous in flower strip plots, whereas Tydeiidae and the predatory mite *Zetzellia mali* (Ewing) were more abundant in grass plots. Densities of the leafminer moth *Leucoptera malifoliella* (O.Costa) were highest in flower strip plots as compared with the other two treatments, although more parasitized in the flower strip plots [75]. High immigration rates may have biased effects of parasitism regulating leafminers. In contrast, flower strips reduced densities of woolly apple aphids. However, the major crop damage was due to *A. orana* and *C. pomonella*, and flower strips did not reduce the infestation level of these two species. There was generally no difference in fruit damage between flower, grass and bare soil treatments. 

As already reported for cover crops, a careful selection of plant species to fit the particular needs of natural enemy groups was an attempt to improve the impact of the floral resources in the orchard (Table 1). Fourteen species of flowering plants were first examined for their attractiveness to beneficial insects in small plot field tests [76]. Then a selected mixture of cornflower (*C. cyanus*), corn marigold (*Chrysanthemum segetum* (L. Fourn.) and corn chamomile (*Anthemis arvensis* L.) was sown in strips under the trees in an apple and a pear orchard in UK [76]. Anthocoridae were more abundant in the tree rows with the flowering strips. Miridae, parasitoid Hymenoptera, spiders and Coccinellidae were present in the flowering strips, but total seasonal counts were similar in flower strip-treated and bare plots. In the pear orchard, the effect on pest suppression was measured using potted pear trees infested with pear psyllids. Their densities declined over time in both treatments to a comparable extent, suggesting that mainly mobile predators contributed to the reduction. Such a tailored approach ([77] in selection of flowering plants was also performed in a recent study, where flower-strips that serve both pollinators (with “concealed nectar” plants) and natural enemies (with “open-nectar” plants) between tree rows were established in four cider apple orchards [78]. The flower mixture with open-nectar plants attracted many natural enemies like aphidophagous species and parasitoid Hymenoptera. Predation on exposed sentinel egg cards was enhanced in all plots with flower strips in comparison with the control. However, the abundance of natural enemies in aphid colonies in the apple trees did not differ and there were no significant effects on fruit quality. 

The full effect of adding floral diversity to an orchard, in terms of reducing insect pests and promoting their natural enemies, can take years to unfold, thus being longer than the duration of most research projects. For instance, from the second to the third year after the establishment of flower strips in orchards in the pan-European study of Cahenzli et al., the number of preadult codling moths in cardboard traps decreased more in the flower strip plots as compared with the control plots, resulting in reduced fruit damage [57]. The authors suggest that the benefits of an established perennial flower strip may be best observed over a more prolonged period. In a five-year study (starting 1992) on the effects of adding perennial flower strips (composed of *Tanacetum vulgare* L., *Chrysanthemum maximum* Ramond, *Aster tongolensis* Franchet, and *Achillea millefolium* L.) to an existing apple orchard treated with pesticides on an as needed basis, the plum curculio decreased nearly to a quarter within two years after sowing flower strips, while it was only halved in the control [56]. In 1995 and later, insecticide treatments were applied and the infestation by this pest remained less than 0.5%. Flower strips also reduced plant bugs significantly, whereas results against apple maggot were inconclusive and no effect was found on apple sawfly. Codling moth and spring and summer Lepidoptera stayed at low and similar densities across treatments, possibly due to secondary effects of chemical treatments. While results were not conclusive for all pests, the effect on yield from 1992 to 1997 demonstrated that the percentage of damaged apples in the experimental block decreased from 95.2% to 9.2%. However, it decreased only from 67.9% to 32.5% in the control block during the same time interval [56]. 

Although pest control through natural enemies’ enhancement by flower strips is not always guaranteed, flower strips offer a promising agri-environmental scheme. In the recent study of Cahenzli et al. [57], it was found that omnivorous Forficulidae and generalist predators (including spiders, Anthocoridae and Miridae) were not as much attracted by the presence of *D. plantaginea* as aphid specialists such as Syrphidae, Chrysopidae, and Coccinellidae. Therefore, the authors suggested that the presence of the important generalists was not dependent on the density of aphids but was enhanced by flower strips. This makes generalist predators ideal targets for habitat manipulation and conservation strategies, because such natural enemies may be attracted to the orchard independently from aphid infestation level and could be important for early predation upon arrival of the pest [57]. 

Based on these findings, carefully selected, perennial flower strips in inter-rows are suitable to increase floral resources in commercial orchards and to achieve better pest control by a range of natural enemies. The general improvement of habitat conditions within the crop system favored especially more resident generalists like spiders, predatory bugs, and earwigs. In contrast, effects of highly mobile Coccinellidae or Syrphidae might have been underestimated by the cited studies. They probably profited from the floral resources, but their ecosystem service (aphid control) was not restricted to the treated plots and probably more directed by prey densities. In the future, more research is needed to focus on the concrete design of such flower strips adapted to the particular situation, pest pressure and pedoclimatic conditions. Using perennial plants including herbs and grasses can ensure a midterm species-rich plant community [70], whereas annual plants are less useful to implement in perennial crops due to their short life span and less robustness to mulching. Wild, native species (ecotypes and wild forms) are better adapted to the local climate than cultivated forms and are more competitive with the spontaneous flora in apple orchards [79,80]. Therefore, seeds of native origin should be used [9]. After establishment, monitoring studies are needed to discover long-term changes of improved fruit health and reward for the farmer. 

### 3.4. Hedgerows 

Hedgerows are perennial multipurpose structures serving as windbreaks and field borders [11]. They can also provide edible fruits and offer important nesting sites for wildlife. They can be monospecific or highly diverse and their age can be up to hundreds of years. Some are intensively managed, cut and kept narrow; others are allowed to grow and can be quite wide, with more annual vegetation at the base. Flowering trees and bushes in hedgerows can provide rich and early season sources of pollen and nectar [12]. Hedgerows provide connectivity to the landscape, and they are sources of plant diversity, flowers and higher microclimate diversity. These qualities make hedgerows important sources of arthropod diversity in the agricultural landscape. Agricultural intensification, with larger farms and larger fields has led to a reduction of hedgerows in the landscape. 

Studies conducted to assess the role of hedgerows for insect pests and their natural enemies in apple orchards demonstrated that the presence of hedgerows provides higher abundance and diversity of arthropods in the orchard [81,82]. The same is true for birdlife [83]. Pollen diversity and abundance was higher on natural than on secondary hedgerows [84]. Generally, pollen densities reached relatively high levels from late April to late June and decreased in summer. Pollen is an important food resource for predatory mite populations, for example *Typhlodromus pyri* Scheuten, in orchards. Certain border plants were identified as sources for predatory mites in apple orchards [85,86]. Studies also showed that presence of hedgerows provide higher density of other predators and pollinators in orchards, leading to numbers of these beneficials being higher near the hedgerow and falling towards the centre of the orchard, whereas lower numbers of pear psylla and overwintering codling moth larvae were found near the hedgerow than away from it [82,87]. Such a positive effect of hedgerows was also shown for codling moth parasitoids, while the opposite was true for their hyperparasitoids [88]. 

Mobility of natural enemies like coccinellids, true bugs, earwigs and syrphids between the hedgerows and the orchards has also been documented [81,82,89,90], showing that different groups/species have different habitat preferences. A high correlation in species composition between hedgerows and orchards supports the migration of syrphids [89,90], but their high mobility makes finding a gradient difficult. ‘Canopy insects’, including the green lacewing *Chrysoperla* sp., in contrast had a preference for the orchard and frequently moved from the hedgerow to the orchard [91]. Spatial aspects of hedgerow orchard dynamics are further linked to wind exposure, with higher psyllid [81] or codling moth larvae [88] densities in parts of the orchard not protected by or further away from the hedgerow. Frequent movements between the orchard and the adjacent hedgerow were recorded for a diverse range of predator taxa (*F. auricularia*, *Chrysoperla* sp., *Philodromus* spp., *Cheiracanthium mildei* L. Koch, and *Nebria brevicollis* (Fabricius)) in Southern France [91]. Regarding hedgerow quality, diversified perennial plants in hedgerows increased the probability of the ‘canopy insects’ like the ground beetle *N. brevicollis* and spiders staying within the orchard and of *Philodromus* spp. and *Chrysoperla* sp. entering the orchard [91]. In addition, thickness of the hedgerow also had an influence on the dispersal of natural enemies: the probability of the ‘ground beetles’ entering and staying in the orchard and probability of the *Philodromus* spp. and *Chrysoperla* sp. entering the orchard was enhanced with higher thickness, probably due to better wind protection [91]. 

These examples confirmed the role of hedges as important source habitat of natural enemies and also demonstrated better pest control in apple trees adjacent to hedgerows. Furthermore, their particular composition can deliver valuable resources for many natural enemies, including important mobile aphid antagonists like Syrphidae and Chrysopidae but also more resident spiders, earwigs, and carabids. The exact role of (early) blooming species of the hedge on beneficials deserves further study in order to elaborate a potpourri of recommendable plant species for different apple growing regions. The impact on the orchard may decrease with its size and is probably limited in the case of expanded plantings. Despite the important encouragement of farmers to consider also the design and the management of hedges around orchards, other connected ecological infrastructures, which act inside the orchard, are also necessary. 

### 3.5. Intercropping and Agroforestry 

The idea of intercropping is to grow two or more crops together in the same field in order to optimize land use and to exploit synergistic effects. In fruit tree cultivation, intercropping can also be considered an agroforestry system, growing fruit trees together in polycultures, with vegetables or even with grazing animals. Brown and colleagues examined the effect of interplanting apple with peach trees, bearing EFN with the aim to improve pest control [92,93,94,95]. In a range of trials, the control of aphids, leafrollers and other moths was examined by sentinel prey exposure and/or assessment of pest and beneficial arthropod densities. The effect varied; even though it was supposed that EFN may contribute to a rapid satiation of beneficials like *Harmonia axyridis* (Pallas), reducing their predation rate on aphids [95], it was found that this lady beetle was probably attracted by EFN, thus arriving earlier in the inter-planted orchard [93]. Lower rosy apple aphid infestation in spite of reduced mortality in aphid colonies, most likely due to ant protection of aphids, was observed in the interplanted orchards. The “resource concentration” hypothesis was suggested to explain these findings, meaning that rosy apple aphids migrating back to host trees for oviposition the previous autumn had been more attracted to monocultures, thus creating higher aphid densities there [92]. Infestations by pentatomids and San Jose scale (*Quadraspidiotus perniciosus* (Comstock)) were more severe in the monoculture than in the diversified system. No higher parasitism of sentinel larvae of the tufted apple bud moth *Platynota idaeusalis* (Walker) was found in the intercropped field, although the longevity of the parasitoid *Goniozus floridanus* (Ashmead) increased when provided with EFN in laboratory trials [94,96]. The same research team reported on trials where the orchard design considered polycultures of fruit tree species (sweet cherry, pear, peach, and apple) with or without companion plants (buckwheat, purple tansy and others) in comparison with monocultures of apple [97]. More Coccinellidae and other predators were observed in the diversified treatments, but pest control was not improved and fruit damage was not reduced. Fruit yield was even highest in monoculture plots. A certain different approach was the intercropping of aromatic plant species (e.g., *Ageratum houstonianum* Mill., *Tagetes patula* L., *Ocimum basilicum* L., *Mentha canadensis* L.) in an organic apple orchard [98,99,100]. In general, herbivores and in particular the spirea aphid (*Aphis citricola* van der Goot = *Aphis spiraecola* Patch) and Tortricidae were less abundant in the intercropped plots. Abundance and diversity of predators and, in [98], also of parasitoids were higher in the diversified plots. The authors suggested the “repellent chemical” hypothesis (i.e., aromatic plants caused a repellent effect to herbivores by their volatile oils, thus preventing successful host plant location) as well as the “natural enemy” hypothesis (i.e., increased pressure by enhanced population of natural enemies) as potential explanations for the altered predator-prey relationship in the intercropped orchard plots. Similar results were found in a comparable study by the same research team in pear [101]. 

Introduction of crop plants with particular traits (e.g., EFN, volatile oils) in the cropping system can deliver options to direct natural enemies and pests in a way that infestations on the main crop are reduced, even simply by increased plant diversification in space. Of course, such interventions often require a forward-looking system design and are not easy to implement, especially not in already existing plantings. More research is needed to design suitable farmers’ systems that namely account for cultural and harvest operations. While there is increased interest in agroforestry systems, research on fruit-tree based agroforestry systems is still scarce, especially in temperate regions. Focusing on mixed vegetable apple orchards, Imbert et al. compared densities of predator arthropods and predation of sentinel aphids on cabbages grown in the inter-rows between apple trees and in plots situated 20 m apart in Southern France. Few differences in predator abundance and predation rates were found in the cabbage plots between and apart from fruit trees. The authors concluded that compared with monoculture vegetable systems, the inter-cropped apple trees might offer alternative prey species that may distract predators from consuming pests in cabbage [102]. 

In contrast to the other topics discussed in this review, intercropping and agroforestry systems are of particular interest by the provision of multiple services, namely production (not only of fruit), improvement of the environment, fertilization and water recycling [103,104]. Reviews and even meta-analyses exist on the benefits of crop plant diversity for pest and disease control and other services [104,105]. But regarding agroforestry, the focus is more on tropical systems [106]. Accordingly, research on major crop systems in temperate regions should be highly encouraged to initiate innovations in crop design. 

## 4. General Conclusions and Further Perspectives

The approaches reviewed to increase plant diversity in the apple orchard ecosystem followed different degrees of intervention: short-term practices (mowing regime and weed maintenance, cover crops), creation of longer lasting ecological infrastructures (perennial flower strips, hedgerows) and intercropping and agroforestry targeting a more profound re-design of cropping systems. Impacts of these approaches act on different levels, starting in the microcosmos of the orchard via growers’ daily practice, up to decisions to be made by stakeholders and politicians. Further consequences of these different levels are proposed as follows. 

### 4.1. Effect on Biodiversity and Ecosystem Services 

We selected studies with the major goal of improving pest control by increasing floral diversity and thus floral resources for nutrition of natural enemies in apple orchards. In general, the enrichment of the vegetation was achieved by all the practices described, regardless of whether introducing annual cover crops or polycultures, for instance apple mixed with peach trees. However, there was no straightforward effect on the third trophic level (antagonists of herbivores). Reasons are certainly manifold, but several major trends can be recognized. The multiple services targeted by floral biodiversity enrichment in early studies led to a rather vague design of the measures of being ‘supportive’ or ‘beneficial’, while later studies have also included more specific exploration of selected elements. An important factor to consider is that most studies are of a short duration of two to three years. The few studies followed over a longer period showed increasing effects with time [56,62], reflecting the long time needed to build up the required ecological processes, including population dynamics of natural enemies. 

The variable outcome or lack of clear conclusions in some of the studies may be due to overlooked elements of species biology. Our knowledge of biology and ecology of pests and natural enemies in relation to floral diversity is still incomplete. Although the nutritional role of non-prey diets such as pollen and nectar for generalist predators is likely more important than assumed [107,108], it is not clear how it may—or may not—complement a predator’s diet. Natural enemies could also benefit from increased functional biodiversity providing overwintering habitats and optimizing the microclimate, but tightly focused research on these topics is needed. Finally, any potential effects of intra-guild competition or the enhancement of “natural enemies of natural enemies” such as hyperparasitoids should also be considered [109]. 

Whereas effects augmenting natural enemies may be difficult to demonstrate, at least in those studies where particular groups of natural enemies (coccinellids, syrphids, and specific parasitoids of key pests) were targeted by selective floral diversity, the hypothesis of increased abundance in such diversified habitats was confirmed. While “tailored” plant mixtures attracted higher numbers of these organisms, sometimes even confirmed as flower visitors ([32,67,78], this response could not always be translated into their improved action as pest regulators. Better pest control by particular antagonists was often shown by use of sentinel prey, although not on natural pest populations. In contrast, the effect of the whole “predatory complex” or even the presence of the ecological infrastructure itself was reported to increase the ecosystem service of pest regulation [56,57] (supporting results found in other crop systems [110]). 

In general, less-mobile organisms (predatory mites, spiders, earwigs, anthocorid bugs) showed a more differentiated response regarding their ecosystem service in enriched orchard (parts) than more mobile, strong flyers (e.g., flies including Syrphidae, Coccinellidae, probably also larger parasitoid Hymenoptera). Flower-rich margins (sown or from spontaneous vegetation) of apple orchards attracted many adult hoverflies, but numbers of their larvae in aphid colonies were related to the aphid colony density, not to the vegetation management at the border of these orchards [111]. Also, in farmland, it was shown that local floral abundance and floral diversity were less important for abundance or species richness of adult aphidophagous hoverflies than local aphid densities, probably due to the capacity of the hoverflies to use resources over a comparatively large space [112]. The degree of landscape complexity may be more relevant for the maintenance of metapopulations of species with high dispersal capacity [113,114]. More resident organisms in the orchard seem to benefit from adjacent “woody” structures like hedgerows as source habitat [91], at least in intensively managed IPM orchards [115]. According to our synthesis, this group of natural enemies also responded well to perennial flower strips and spontaneous vegetation within the orchard. But, in a typical monoculture district with a high orchard cover in the landscape, the overall richness of beneficial arthropods is low and it might be very difficult to increase it with any ecological infrastructure [6]. We excluded studies on landscape effects in our review, since we targeted approaches that could be implemented by individual farmers in their orchard following their own decision. Yet—to conclude—the management of plant diversity should not be limited exclusively to a specific scale, because probably a multiscale approach is required, in which temporal and spatial diversification are integrated at all different scales [16,116]. 

Of the 66 studies analyzed here, only 16 included measurement of fruit damage, fruit health at time of harvest, or yield gain or loss for the farmer. However, in terms of interest for the farmer, these data are necessary to evaluate the purpose of such measures for fruit growing and to convince growers about their adoption and implementation on a long term. This was also concluded by a general review, in which pros and cons of flower strip management for farmers were discussed [117]. In addition, potential disservices such as increased levels of some insect pests, diseases or voles [54,55,72,118] when adopting “living mulches” need to be considered. Such studies will allow calculating the tradeoff as a basis for farmers’ decisions to introduce or abandon any practice. 

### 4.2. Effect on Orchard Management 

Based on the results of a questionnaire of growers and advisors (mainly, but not exclusively organic fruit production) that was conducted in several European countries (France, Germany, Denmark, Sweden, Switzerland, Latvia, Italy) within the framework of the research project “EcoOrchard”, we could confirm the European-wide interest and motivation of growers to increase functional biodiversity in their crop system. Different approaches are already common practice and growers consider these techniques as a toolbox to enhance multiple ecosystem services in addition to pest regulation [119]. 

**Figure 3:**
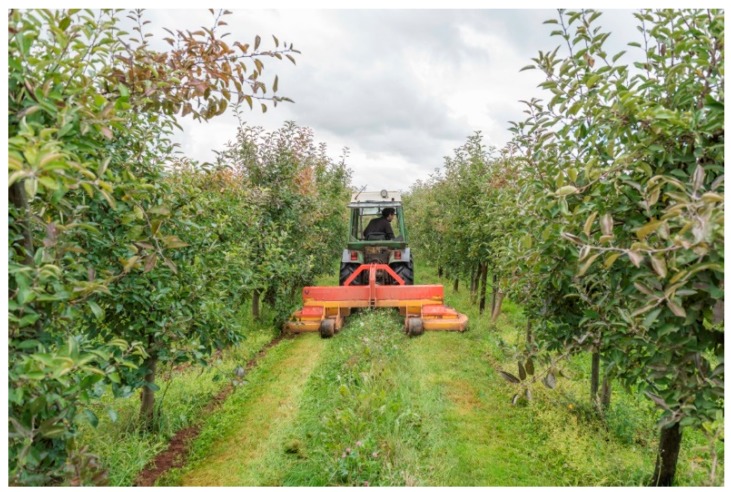
Management of inter-row perennial flower strips in an apple orchard by using a specific mulching device. The flower strip is about 0.5 m wide, whereas the adjacent vegetation is cut down frequently to reduce establishment of voles and competition for water and nutrients. Foto: Simon Feiertag, JKI Darmstadt ©.

As an outcome of our review, it became evident that the establishment of selected, perennial flower strips in inter-rows may be a promising technique for commercial orchards. Also the cautiously managed growing of spontaneous vegetation in inter-rows deserves further study, because it may have a similar effect and can be actively manipulated by the farmer. For instance, the frequency as well as the mulching height influence plant diversity in perennial flower strips as well as in the spontaneous vegetation cover [70]. But mulching may also reduce the growth of unwanted weeds in flower strips [35]. Mowing in early summer can result in higher cover of target species and less competition by grasses as compared with mowing in autumn, as shown in a study in grassland [120]. However, an excessively reduced mowing regime can also lead to an increase in grasses and ultimately to a reduction in plant diversity [120]. For a successful implementation of flower resources in the midterm, it is therefore crucial to apply a site-adapted mulching intensity [70]. Moreover, frequency and timing of mulching flower strips must be carefully aligned with the stage of development of beneficial arthropods so that they will not be physically harmed and their habitat and food resources will remain intact [121]. The right time for mulching needs to be decided by observing the population cycle of beneficial arthropods over the years or by using available data on life cycles [122]. In our pan-European study, we found that intensive mulching of flower strips greatly affected the plant community and decreased species richness and ground cover by forbs and plants, which especially promote functional biodiversity [70]. Specific machinery is available in some European countries for the management of flower strips and adjacent parts of ground cover and may help the grower to reduce workload (Figure 3). 

One major important aspect to consider when promoting functional biodiversity in orchards is an adapted regime of the use of plant protection products. Especially in integrated production, considerations are needed regarding when and how to apply pesticides and how to avoid any negative effect on flower visitors or non-pest inhabitants of the flower strips. In this regard, the development of specific guidelines including the provision of thresholds for the necessary ratio of natural enemies to pests for natural regulation could greatly limit the intervention with insecticides, thereby enhancing the activity of beneficials. In general, decisions to apply plant protection products need to be based on a careful monitoring of pest species and their natural enemies and only used when necessary. Recommendations for use of plant protection products also have to incorporate the degree of implemented ecological infrastructures and how to prevent negative side-effects by proper timing and careful selection of the active ingredients used. Environmentally safe top-down control methods such as mating disruption [123] or endured efficacy of the codling moth granulovirus as a selective biopesticide [124] are expected to further promote natural pest control, because of the lower disturbance due to a reduced use of nonspecific insecticides. An increased uptake of such solutions by conventional farmers also is highly desired. 

Additional innovative approaches including non-crop vegetation hold great potential for sustainable apple production. The involvement of EFN-bearing plants belongs to such a concept [46]. EFN-bearing plants are not only interesting because they feed natural enemies. They are also involved in the interaction between ants and aphids and—by increasing the availability of alternative plentiful sugar sources—may reduce ant-tending of aphid populations in the main crop. Such plants can be included in the orchard system as cover crops, in flower strip mixtures and as perennial shrubs or fruit trees in agroforestry or hedges as well. Nagy et al. studied an alternative method for the manipulation of the complicated relationship between aphids, tending ants and sugar sources [125]: instead of introducing plants with EFN, feeding stands offering sucrose solutions to ants (artificial nectaries) were exposed. The significant reduction in aphids was explained by a distraction of tending ants and thus an increased predator pressure on the pest. Another example is the combination of flower strips with herbivore-induced plant volatiles. Such approaches need to be investigated, since positive interactions among two such components are likely to emerge, as shown in other cropping systems [126]. 

Connectivity between ecological infrastructures, namely between perennial flower strips and hedges, and between flower strips and the tree canopy is crucial, especially in expanded fruit growing regions. Future research effort should include studies on suitable orchard design and may also be elaborated together with practitioners [119]. 

### 4.3. Considerations for Stakeholders and Policy

Approaches to create and increase local (functional) biodiversity in perennial crops can help to improve pest regulation, thus contributing to more environmentally safe pest management. Moreover, their successful implementation can also contribute to combat biodiversity loss and insect decline, not only at the local scale, but also at the landscape level, especially if applied by several growers in cooperative actions in region-wide areas of fruit cultivation. The concept should also be transferred to other crop systems; e.g., stone fruit or nut orchards, berries or vineyards. In addition, it is necessary to explore the real required input costs (material, work load, risk of unforeseen pest encouragement etc.) in order to judge potential profit or loss perspectives [127]. One important step would be to include such measures as soon as possible in agri-environmental schemes and thereby motivate and acknowledge farmers for their adoption. 

## Figures and Tables

**Table 1:** Plant species introduced as cover crops, flower strips or intercrops to enhance pest control in apple orchards. Spontanous vegetation (weeds) is not considered.

Family	Species	Reward System	Flower Type [128]	Managed As	Reference
Apiaceae	*Ammi majus* L.	Nectar, pollen	Disk flowers with nectar open	Cover crop, flower strip	[54,78]
Apiaceae	*Anethum graveolens* L.	Nectar, pollen	Disk flowers with nectar open	Cover crop, flower strip	[36,78,97]
Apiaceae	*Carum carvi* L.	Nectar, pollen	Disk flowers with nectar open	Flower strip	[57,71,72]
Apiaceae	*Coriandrum sativum* L.	Nectar, pollen	Disk flowers with nectar open	Cover crop, flower strip	[28,41,78]
Apiaceae	*Daucus carota* L.	Nectar, pollen	Disk flowers with nectar open	Flower strip	[57,60,66,72,78]
Apiaceae	*Foeniculum vulgare* Mill.	Nectar plentiful, pollen	Disk flowers with nectar open	Cover crop, flower strip	[54,72,78]
Apiaceae	*Pastinaca sativa* L.	Nectar plentiful, pollen	Disk flowers with nectar open	Flower strip	[60,78]
Asteraceae	*Achilea millefolium* L.	Pollen, nectar	Flower heads, ray and disk flowers	Cover crop, flower strip	[54,56,67,69,78]
Asteraceae	*Cichorium intybus* L.	Pollen, nectar	Flower heads, only ray flowers	Cover crop, flower strip	[54,57,60,78]
Asteraceae	*Leucanthemum vulgare agg.*	Pollen, nectar	Flower heads, ray and disk flowers	Flower strip	[57,60,71,72]
Asteraceae	*Matricaria chamomilla* L. *(M. recutita* L.*)*	Pollen, nectar	Flower heads, ray and disk flowers	Flower strip	[60,72]
Asteraceae	*Tanacetum vulgare* L.	Pollen, nectar	Flower heads, only ray flowers	Flower strip	[56,78]
Brassicaceae	*Brassica napus*, *Sinapis alba*, *Sinapis arvensis*	Nectar open, pollen	Disk flowers with nectar open	Cover crop, flower strip	[36,54,72]
Brassicaceae	*Lobularia maritima (*L.*)*	Pollen, nectar	Disk flowers with nectar ± hidden	Cover crop, flower strip	[40,53,78]
Boraginaceae	*Phacelia tanacetifolia* Benth.	Nectar, plentiful	Funnel flowers, corolla tube long	Cover crop, flower strip	[38,40,78,97]
Caryophyllaceae	*Silene vulgaris (*Moench*)*	Nectar	Stalk disc flowers, stamina and pistil outside tube	Flower strip	[69,71]
Dipsacaceae	*Knautia arvensis (*L.*)*	Nectar, pollen	Flower heads	Flower strip	[60,72,78]
Fabaceae	*Lotus corniculatus* L.	Nectar, pollen	Flag blossom	Flower strip	[57,60,72,78]
Fabaceae	*Medicago sativa; Medicago lupulina* L.	Nectar, pollen, extrafloral nectaries?	Flag blossom	Cover crop, flower strip	[37,44,50,51,57,60,72,78]
Fabaceae	*Trifolium repens* L., *Trifolium fragiferum* L., *Trifolium sp.*	Pollen, nectar and extrafloral nectaries?	Flag blossom	Cover crop, flower strip	[39,72,78,129]
Fabaceae	*Trigonella foenum-graecum* L.	Pollen, nectar	Flag blossom	Cover crop	[54]
Fabaceae	*Vicia faba L.*, *Vicia sativa*, *Vicia dasycarpa*, *Vicia cracca*, *Vicia sepium*	Pollen, nectar hidden, long corolla, extrafloral nectaries	Flag blossom	Cover crop	[42,49,57,72,78,129]
Polygonaceae	*Fagopyrum esculentum* Moench	Pollen, nectar open, plentiful	Disk flowers with nectar open	Cover crop, flower strip	[36,38,40,42,43,54,78,97]
“Aromatic plants”, mainly Lamiaceae	*Mentha canadensis*, *Ageratum houstoniuma*, *Ocimum basilicum*, *O. citriodourm*, *Nepeta cataria*, *Tagetes patula*, *Satureja hortensis*	Pollen, nectar, Volatiles (repellent?)	Various, depending on species	Intercrop	[98,99,100,101]
Rosaceae	*Pyrus communis* L., *Prunus avium* L., *Prunus persica (*L.*)* Batsch	Pollen, nectar, extrafloral nectaries	disk flowers with nectar ± hidden in centre of flower	Intercrop	[92,93,94,97,130]

## Supplementary Materials

The following are available online at https://www.mdpi.com/2075-4450/10/8/247/s1: Table S1: Adaptation of Mowing Regime and Weed Maintenance; Table S2: Cover Crops; Table S3: Annual and Perennial Flower Strips; Table S4: Hedgerows; Table S5: Intercropping.
